# Follow-up imaging after hospital discharge for acute intracerebral hemorrhage in European academic hospitals: a decision-making analysis

**DOI:** 10.1007/s00234-026-03927-8

**Published:** 2026-03-04

**Authors:** Gonçalo G. Almeida, Jan Liebelt, Paul Martin Putora, Johannes Weber, Simon Wildermuth, Sebastian Leschka, Tobias Johannes Dietrich, Pasquale Mordasini, Franca Wagner, Meike W. Vernooij, Kamil Zeleňák, Alexandre Krainik, Fabrice Bonneville, Luc van Den Hauwe, Markus Möhlenbruch, Federico Bruno, Birgitta Ramgren, Ana Ramos-González, Till Schellhorn, Stephan Wälti, Tim Steffen Fischer

**Affiliations:** 1 Department of Radiology, Kantonsspital Baden, affiliated Hospital for Research and Teaching of the Faculty of Medicine of the University of Zurich, Baden, Switzerland; 2 University of Zurich, Zurich, Switzerland; 3 Department of Radio-Oncology, HOCH-Schweiz, St. Gallen, Switzerland; 4 University of Bern, Bern, Switzerland; 5 Department of Radiation Oncology, University Hospital of Bern, Bern, Switzerland; 6 Zentrum für Neuroradiologie, Klinik Hirslanden, Zurich, Switzerland; 7 Department of Radiology and Nuclear Medicine, HOCH, Cantonal Hospital St.Gallen, St. Gallen, Switzerland; 8 Department of Diagnostic and Interventional Neuroradiology, Cantonal Hospital Aarau, Aarau, Switzerland; 9 Department of Radiology and Nuclear Medicine, Erasmus MC, Rotterdam, Netherlands; 10 Department of Radiology, Comenius University´s Jessenius Faculty of Medicine and University Hospital, Martin, Slovakia; 11 Department of Neuroradiology, University Hospital of Grenoble, Grenoble, France; 12 Department of Neuroradiology, Toulouse University Hospital, Toulouse, France; 13 Department of Radiology, Antwerp University Hospital, Antwerp, Belgium; 14 Department of Neuroradiology, University Hospital Heidelberg, Heidelberg, Germany; 15 Department of Biotechnology and Applied Clinical Sciences, University of L´Aquila, L’Aquila, Italy; 16 Diagnostic Radiology, Department of Clinical Sciences, Lund University, Lund, Sweden; 17 Department of Neuroradiology, University Hospital Madrid, Madrid, Spain; 18 Department of Radiology and Nuclear Medicine, Oslo University Hospital, Oslo, Norway; 19 Department of Radiology, Kantonsspital Winterthur, Winterthur, Switzerland

**Keywords:** Intracerebral hemorrhage, MRI, MR-Angiography, CT-Angiography, Digital subtraction angiography, Decision-making analysis, Consensus, Small-vessel disease, European academic centers

## Abstract

**Purpose:**

To investigate factors guiding follow-up imaging after hospital discharge in atraumatic intracerebral hemorrhage (ICH) across large European academic centers and compare decision-making patterns using a standardized approach.

**Methods:**

Expert neuroradiologists from nine European centers in eight countries detailed their follow-up algorithms for deep and lobar ICH, specifying (1) patient-related decision criteria, (2) preferred imaging modalities, and (3) advised follow-up timing. Free-text and schematic inputs were harmonized into uniform decision trees, mapping recommended modalities (CT, MRI/MRA, DSA, CTA) to patient-factor constellations. The primary endpoint was the distribution of recommended strategies; the secondary endpoint was inter-center consensus (≥ 50% agreement).

**Results:**

Key decision criteria were hemorrhage location, suspected structural macrovascular pathology (SMVP), age, hypertension, and small-vessel disease. MRI/MRA was recommended by all centers and appeared in nearly all decision branches; “no further imaging” was rare. Additional strategies variably combined MRI/MRA with DSA and/or CTA, whereas stand-alone DSA as first follow-up test was uncommon. Formal consensus was achieved in a minority of clinical scenarios, most consistently for deep ICH, where MRI/MRA reached around 60% support. In young patients with lobar ICH and suspected SMVP, no single strategy reached consensus. The most frequently proposed timepoint for follow-up imaging was 6–12 weeks after discharge.

**Conclusion:**

In current European practice, MRI/MRA at 6–12 weeks represents the predominant follow-up strategy after ICH discharge, with CTA ± DSA added when SMVP is suspected. Despite this MRI-centered pattern, substantial inter-center heterogeneity persists, underscoring the need for standardized follow-up imaging with clearly defined timelines and protocols.

## Introduction

Intracerebral hemorrhage (ICH) accounts for approximately 9–27% of all stroke events globally and is associated with substantial morbidity and mortality [1, 2]. Typically, ICH are classified based on anatomical location into either deep or lobar hemorrhages, reflecting distinct underlying pathophysiologies [3, 4]. Deep ICH are predominantly attributed to hypertensive vasculopathy, whereas lobar hemorrhages are often linked to cerebral amyloid angiopathy or underlying structural pathology such as arteriovenous malformations or tumors [5].

While acute-phase imaging strategies during hospital stay have been previously analyzed - including in a recent multicenter decision-making study [6] - less is known about follow-up imaging after hospital discharge. Given that imaging modalities often play a central role in therapeutic approach and subsequent patient recovery, uniformizing the standard of care could better guide and support evidence-based decision-making and therefore contribute to better patient outcomes. Follow-up imaging emerges as vital in clinical practice for multiple reasons which include detection of residual hematoma; new hemorrhagic foci, re-bleeding, and delayed complications such as hydrocephalus, vasospasm, ischemia and seizures; monitoring resorption; imaging correlation to functional outcomes; identifying underlying etiologies like vascular malformations or amyloid angiopathy; and informing secondary prevention and rehabilitation planning.

Population-based studies have demonstrated that survivors of atraumatic ICH remain at considerable risk of recurrent cerebrovascular events long after hospital discharge, with both ischemic stroke and recurrent hemorrhage occurring at clinically relevant rates [7]. Long-term cohort data further confirm that this elevated hazard persists for years, indicating that follow-up strategies cannot be limited to the acute hospitalization period alone but rather require structured surveillance across the continuum of care [8]. Importantly, recurrence risk is strongly phenotype-dependent: patients with cerebral amyloid angiopathy (CAA)–related lobar hemorrhage face an annual recurrence of approximately 7–8%, far exceeding the rates seen in hypertensive deep ICH [9, 10], which argues for closer clinical and imaging surveillance in this subgroup. The observation that survivors are at roughly equal long-term risk of ischemic and hemorrhagic events underlines the role of timely follow-up neuroimaging in differentiating new neurological symptoms and guiding secondary prevention measures [7].

The majority of existent guidelines focus on acute care, but recommendations for follow-up imaging timing, modality, and purpose - detection of rebleeding, hydrocephalus, delayed ischemia, long-term sequelae, recurrence risk stratification - are less standardized. Namely, clinical guidelines from both the European Stroke Organization (ESO) and the American Heart Association/American Stroke Association (AHA/ASA) advocate for the utilization of non-contrast-enhanced computed tomography (NECT) as the initial imaging modality for diagnosing acute ICH [11, 12]. The use of advanced imaging modalities such as CT angiography (CTA), MR-Angiography (MRA), or digital subtraction angiography (DSA) during hospital stay is recommended when clinical or radiological factors suggest potential underlying structural vascular abnormalities [13, 14]. The lack of official recommendations for imaging follow-up after hospital discharge renders decision in clinical practice heterogeneous, furthered by the various constellations that can present such as hemorrhage location, patient age, comorbidities, available imaging modalities and resource allocation.

To address this knowledge gap, the primary aim of this study was to determine how neuroradiology experts approach imaging recommendations after hospital discharge in patients who experienced an ICH, and to subsequently analyze the influence of various clinical factors in the decision-making process.

## Materials and methods

**Fig. 1:**
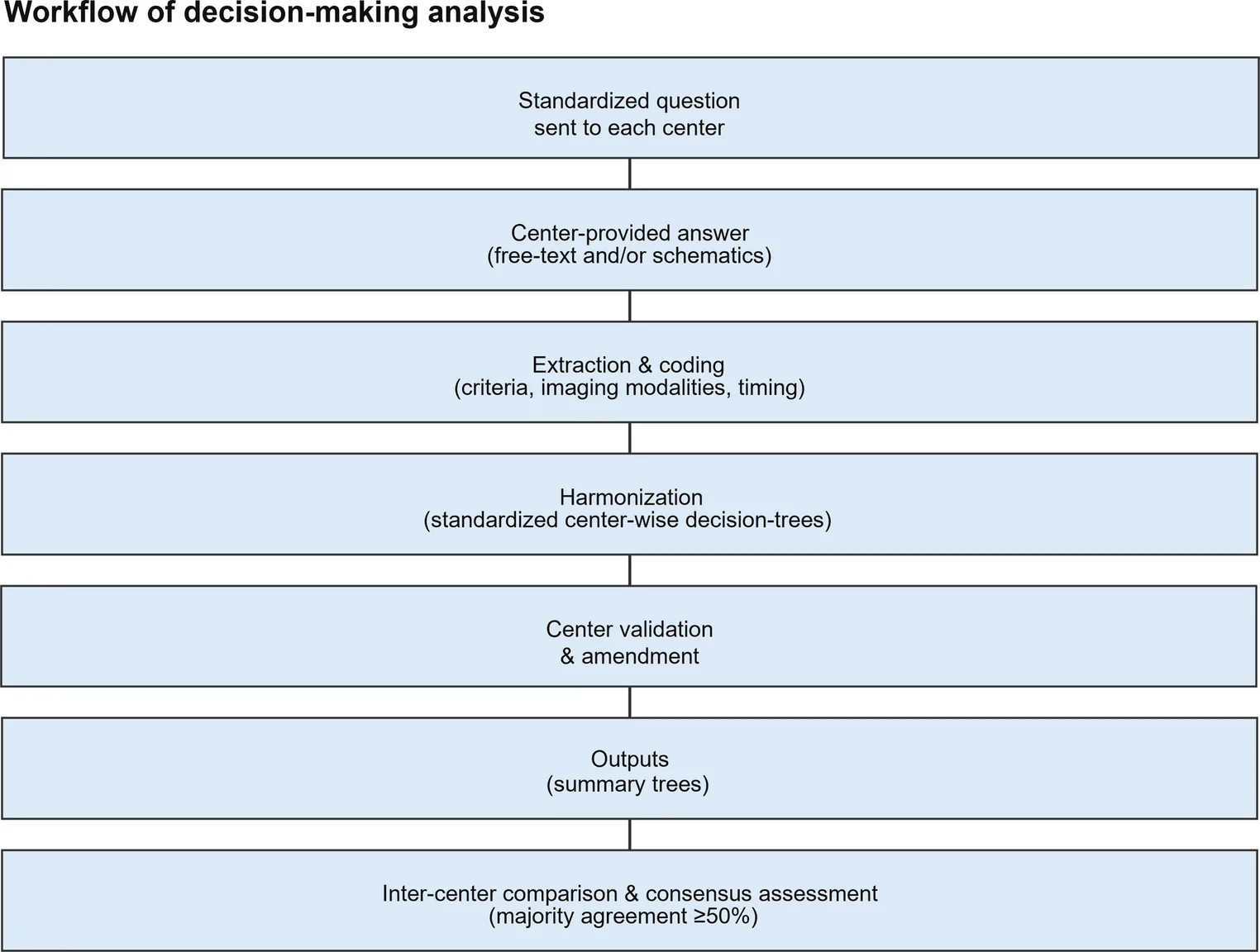
Workflow of decision-making analysis from raw expert input to inter-center comparison and consensus assessment of decision trees

Expert neuroradiologists from large European academic hospitals nominated by the executive committee of the European Society of Neuroradiology (ESNR) were asked to participate in a structured decision-making analysis (Fig. 1). A total of 9 experts in eight countries agreed to participate in the current study (Belgium, France, Germany, Italy, Netherlands, Norway, Slovakia, Spain, and Sweden) and provided answers to the following question:

“In a patient with an atraumatic intracerebral hemorrhage not requiring surgery, investigated during hospital stay (with CT/CTA, MRI/MRA and DSA) without identified cause:

- Which follow-up, if any, would you recommend upon discharge for two given locations: (1) deep hemorrhage [Basal ganglia, thalamus, pons or cerebellum] or (2) lobar hemorrhage?
- Please specify decision-criteria impacting your choice and their influence on potential imaging follow-up post-discharge from the hospital.”

**Fig. 2:**
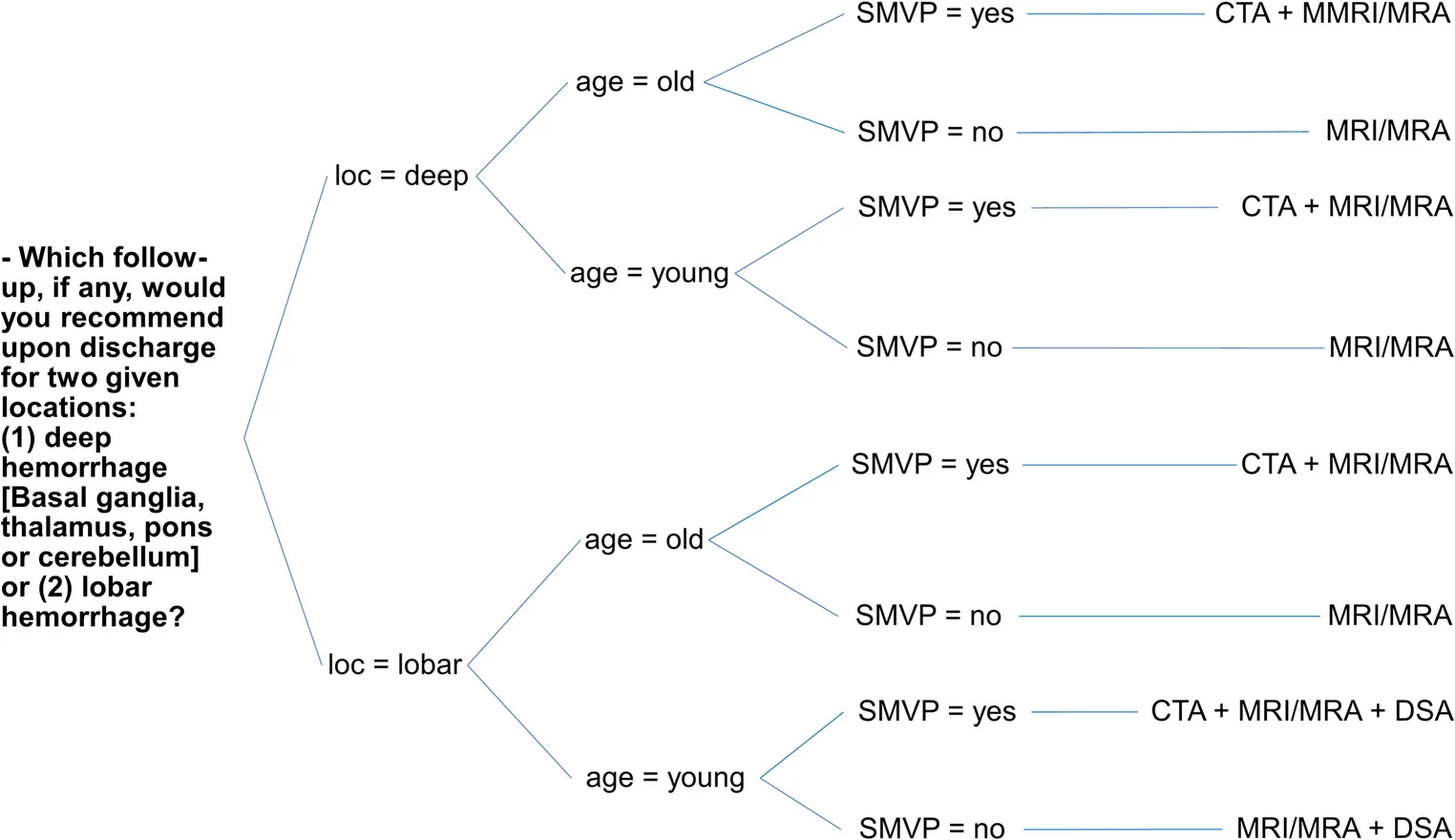
Example of a decision-tree built from the recommendations received from center H

Responses were provided between December 2023 and March 2025 as free text and/or schematic algorithms and converted into decision-trees according to reported criteria (Fig. 2). Criteria reported included “Age” (dichotomized as “young/old”, according to center practice), “Location” (deep vs. lobar), “SMVP” (suspected structural macrovascular pathology - e.g., aneurysm, arteriovenous fistula, arteriovenous malformation), “Hypertension” (HT; present/absent), and “SVD” (imaging signs of small-vessel disease; present/absent). “Age” was analyzed as a binary category even though some centers specified thresholds for “old” between > 50 and > 70 years, whereas others simply distinguished “young” from “old” without a clear cut-off. As such, age was used as a center-defined relative category reflecting local practice rather than a single threshold. Even though the most common reported cut-off for hypertension was systolic blood pressure > 140 to > 150 mmHg, definitions varied across participating centers. As such, hypertension was simplified was a criterion into either present or absent. Isolated criteria reported by only one center were advanced to cross-center analysis only when required to maintain continuity with our previous decision-making study [6, 15]. This was the case for only SVD. The presence of cerebral amyloid angiopathy (CAA) and clinical causes (e.g., drug abuse) did not meet this condition and were therefore omitted from the comparative analysis. Follow-up imaging recommendations included non-contrast CT (NECT), CT angiography (CTA), MRI with MR-Angiography (MRI/MRA) and digital subtraction angiography (DSA), either in isolation or in combination with one another. Follow-up imaging timing was mentioned by only some of the centers (5/9) with the most often mentioned timepoint being at 6–12 weeks after patients’ discharge. However, some centers did not provide a specified timeframe for follow-up imaging, while still advocating for the need for follow-up controls. Draft decision-trees were returned to each center for validation and amendment [16, 17].

After validation, center inputs and individual imaging recommendations were compared and the level of consensus was assessed [18]. Consensus was defined preemptively as a single recommendation supported by ≥ 50% of centers as to represent majority agreement while remaining consistent with prior decision-making analysis methodology [6, 16]. Outputs were prepared as center-wise comparison “trees”.

## Results

Nine European academic centers provided validated, center-level algorithms for post-discharge follow-up imaging after atraumatic ICH. Factors influencing imaging follow-up for each center are depicted in Table 1 for lobar ICH and in Table 2 for deep located ICH.

**Table 1:** Lobar ICH. Decision-making criteria raised by each center that were considered relevant for reaching a recommendation regarding follow-up imaging of Lobar ICH. All criteria were raised individually by each center. A predefined list was not provided. As an example, “Age” and “SMVP” were the main criteria factored into the decision-trees (4/9 and 6/9 centers, respectively). “Hypertension”, as well as “SVD” were not considered by any of the centers when deciding post Lobar ICH follow-up. Given CAA was mentioned as a decision criterion in merely 1/9 centers (Center C), it was not depicted in this table. SVD (Small vessel Disease), SMVP (Structural macrovascular Pathology).

Lobar ICH									
	A	B	C	D	E	F	G	H	I
Age	X				X	X		X	
Hypertension									
Location	X		X			X		X	
SVD									
SMVP	X		X	X		X	X	X	

**Table 2:** Deep ICH. List of decision-making criteria raised by each center that are considered relevant for reaching a recommendation regarding the imaging workup of a deep ICH post hospital-discharge. Akin to the results shown in Table 1, in Table 2 both “Age” (5/9) and “SMVP” (4/9) were the predominant chosen criteria by the various centers in the follow-up imaging decision-making, followed by “Hypertension” (2/9). Analogous to Table 1 results, “SVD” was the least mentioned criterion (1/9) by any of the centers as weighing in the decision for post ICH neuroradiological workup. “Clinical causes” such as “drug abuse” were solely mentioned by center C as a decision factor and therefore not included in this table, as was the case verified in Table 1 for “CAA”. SVD (Small vessel Disease), SMVP (Structural macrovascular Pathology)

Deep ICH									
	A	B	C	D	E	F	G	H	I
Age	X		X		X	X		X	
Hypertension	X					X			
Location	X		X			X		X	
SVD	X								
SMVP				X		X	X	X	

**Fig. 3:**
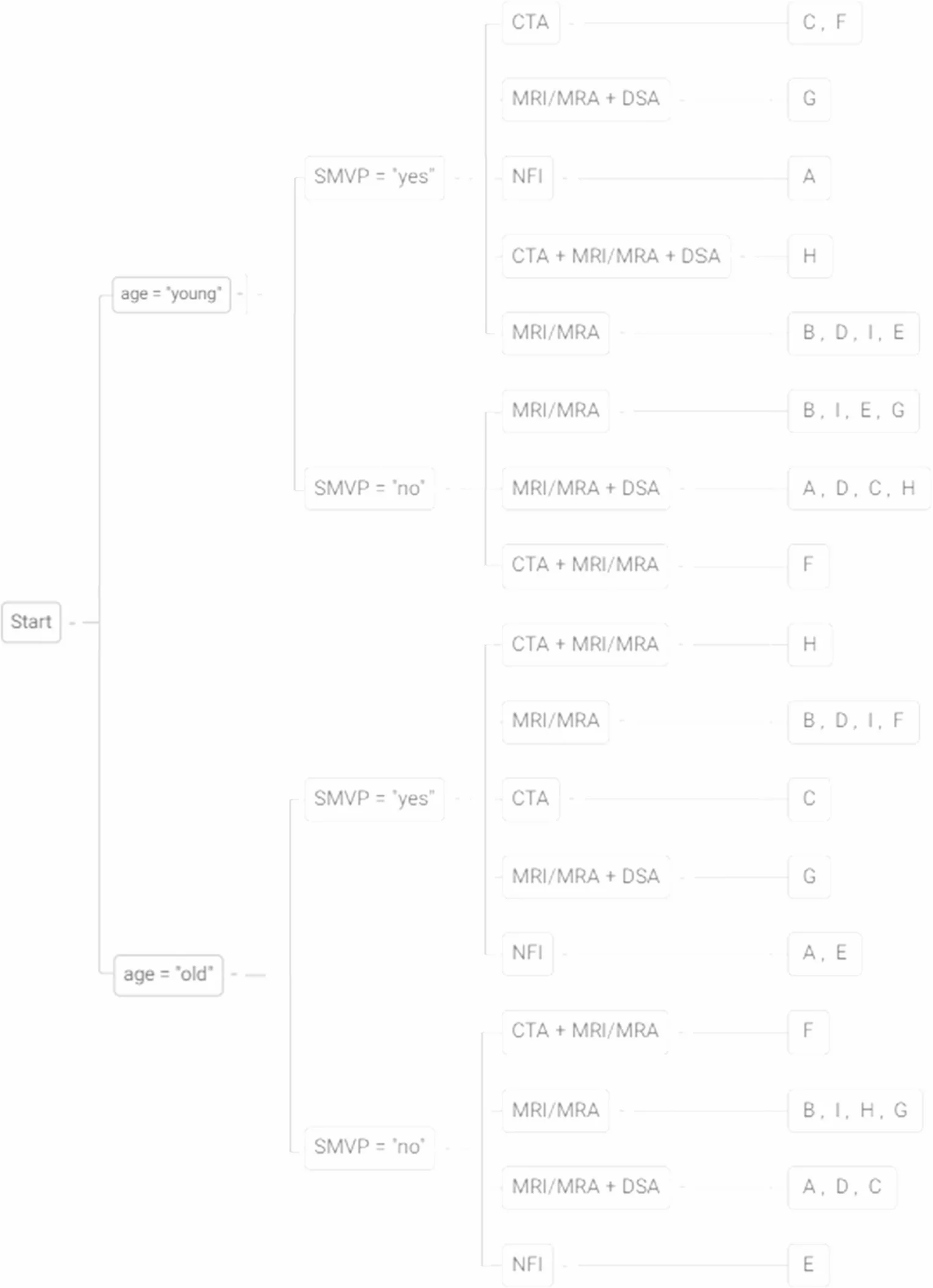
Example of a decision-making algorithm for lobar ICH from multiple centers that uses “Age” and “SMVP” as first and second order criteria respectively, for post hospital-discharge neuroimaging follow-up. As verified in this decision-tree, when faced with patients discharged after lobar ICH, the majority of centers suggested MRI/MRA either alone or in combination with DSA as the most common imaging follow-up recommendation. This mirrors the data shown previously on Table 3. SVD (Small Vessel Disease), SMVP (Structural Macrovascular Pathology)

ICH location was a decision criterion used by all centers (9/9). SMVP was mentioned by the majority of centers (6/9) and just over half of experts mentioned age as a decisive criterion (5/9). HT was solely used by two centers, and SVD was mentioned by a single center. Follow-up imaging recommendations for each center are summarized in Table 3. Imaging strategies recommended irrespective of branch position were dominated by MRI/MRA (9/9 centers). More seldom referred imaging recommendations included MRI/MRA + DSA (4/9), CT native (3/9), CTA + MRI/MRA + DSA (2/9) and CTA + MRI/MRA (2/9). Isolated CTA and DSA were the least recommended imaging methods (1/9 each). “No further imaging” (NFI) was an uncommon recommendation (2/9) and more prevalent in constellations with older patients. Center-wise comparison trees (Fig. 3) highlight this heterogeneity: in cases of deep ICH, MRI/MRA was the most prevalent recommendation, predominantly where SMVP = no. In lobar ICH, a combination of imaging modalities was more frequently reported.

**Table 3:** Imaging Recommendations. List of all recommended imaging technics for post hospital-discharge workup after ICH. It showsa clear prevalence of MRI/MRA either as standalone examination or in combination with otherimaging tools

Recommendation	A	B	C	D	E	F	G	H	I
No Further Imaging Follow-up	X				X				
CT native	X		X			X			
CTA alone			X						
MRI/MRA	X	X	X	X	X	X	X	X	X
CTA + MRI/MRA						X		X	
MRI/MRA + DSA	X		X	X			X		
CTA + MRI/MRA + DSA						X		X	

## Imaging recommendations consensus – Lobar ICH

**Fig. 4:**
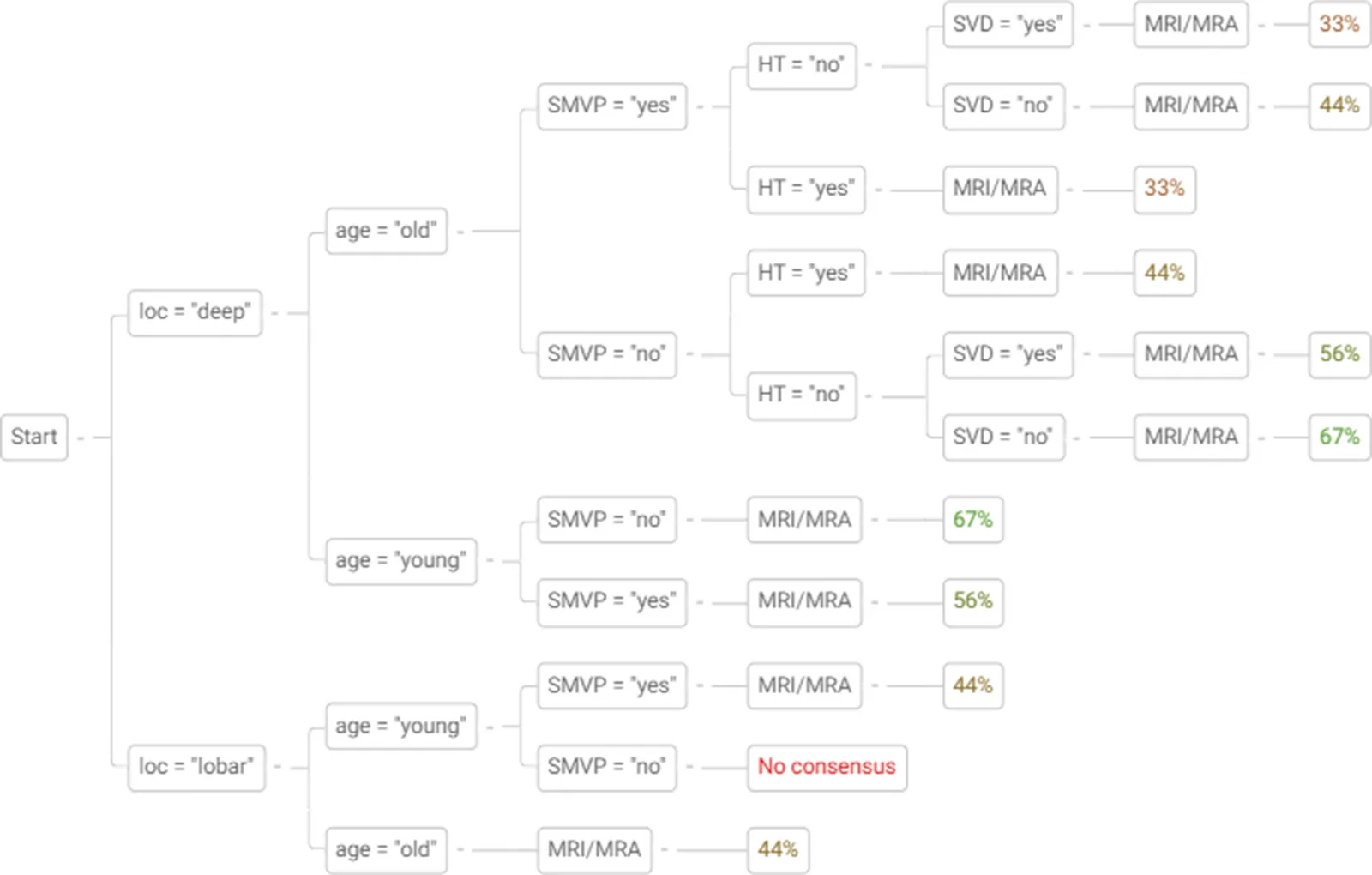
Inter-center consensus variability regarding post hospital-discharge imaging recommendations following ICH. It shows a decision-tree excerpt where MRI/MRA reached a majority agreement (56–67%) among imaging techniques across centers, regardless of first order criteria. However, lower levels of consensus (< 50%) can be observed for MRI/MRA in some particular cases, with no apparent tendency/pattern. On the opposite extreme, for the case of “lobar ICH” in a “young” patient presenting with no “SMVP”, no consensus was verified. SVD (Small Vessel Disease), SMVP (Structural Macrovascular Pathology)

The consensus analysis (Fig. 4) yielded MRI/MRA as the most frequent recommendation in nearly all permutations. Formal consensus (≥ 50%) was reached in a minority of scenarios. For young patients with deep ICH, MRI/MRA was the main recommendation for most centers (5/9–6/9 centers, i.e., 56–67%). For young patients with lobar ICH and SMVP = no, no consensus was reached, owing to a split between MRI/MRA alone and a combination of modalities, namely CTA + MRI/MRA and MRI/MRA + DSA. In older patients presenting with lobar ICH, MRI/MRA was again the most prevalent imaging recommendation in 4/9 centers (44%). There was more variability among older patients with deep ICH: whilst MRI/MRA remained the most prevalent recommendation, consensus was only reached in the specific setting of SMVP = no, HT = no and SVD = no. Where disagreement occurred, it was mainly between MRI/MRA alone and CTA + MRI/MRA. DSA was only rarely recommended in combination with MRI/MRA and never as a stand-alone test.

### Decision-tree example – lobar ICH

Imaging timing preferences were mentioned heterogeneously among centers. The most consistently mentioned interval was at 6–12 weeks.

## Discussion

Across large European centers, for either deep or lobar ICH, MRI/MRA at 6–12 weeks emerged as the prevailing follow-up imaging method after discharge for non-surgically managed spontaneous ICH of unknown cause. CTA + MRI/MRA, MRI/MRA + DSA and NFI were recommended much more rarely. Stand-alone DSA was never recommended as a stand-alone initial follow-up test.

These post-discharge recommendations expand upon our prior admission-phase comparison by showing a pivot toward MRI-centered follow-up once the hyperacute diagnostic window has passed and thus emphasizing the importance of etiological clarification and secondary prevention [6, 12]. This aligns with the rationale that interval hematoma resorption increases conspicuity of occult lesions and that non-invasive vascular imaging can be weighted with the probability of underlying macrovascular pathology. Interval regression of hematoma mass effect and hemoglobin metabolism may reveal parenchymal sources of hemorrhage (e.g., cavernous malformations, hemorrhagic neoplasms) that would otherwise be masked in the acute setting. MRI also allows for further characterization of the brain parenchyma, such as signs of SVD, cerebral microbleeds and cortical superficial siderosis. Susceptibility-weighted imaging (SWI) or T2*-GRE substantially increase detection of cavernous malformations, with SWI outperforming GRE in sensitivity and lesion conspicuity [18, 19].

In cases where macrovascular pathology was suspected (SMVP = yes), a complementary combined imaging approach of CTA + MRI/MRA and/or MRI/MRA + DSA was more often reported. In our experience, CTA tends to be used to address macrovascular lesions with near-term therapeutic implications, while MRI/MRA is used to clarify parenchymal/venous differentials. DSA tends to be reserved for discordant, equivocal, or persistently high-risk scenarios after non-invasive imaging. Evidence from a prospective study corroborates this imaging sequence: early CTA detected a substantial fraction of macrovascular causes, while sequential MRI/MRA and DSA added further diagnostic yield, particularly for cavernomas which were recognized as the hematoma resolves [14].

Our results demonstrate a high level of heterogeneity, as various clinical factors are weighed differently across imaging modalities. Furthermore, these differences are likely accentuated by country-level variation across Europe, including access to outpatient MRI, availability of timely neurointerventional services for DSA, and limited standardization of post-discharge follow-up. These factors influenced center-level decision-making and may also limit the transferability of certain recommendation pathways beyond tertiary academic settings.

Whenever mentioned, imaging follow-up intervals were more often reported at 6–12 weeks, albeit with high heterogeneity. One center advocated for earlier follow-up in 2–6 weeks with CTA + DSA in cases of lobar ICH in young patients with suspected SMVP, possibly so as not to delay therapy. This variance may be in line with the assumed pre-test probability of macrovascular etiology for ICH, which would be consistent with prediction tools such as DIAGRAM, which use age, location, and CTA findings to stratify macrovascular risk and triage the need for invasive angiography [12, 20]. The more frequently proposed 6–12 week follow-up window likely reflects a pragmatic balance between allowing sufficient hematoma resorption to unmask occult lesions whilst avoiding undue delay in treatable macrovascular pathology. This may explain why earlier vascular imaging was proposed in select cases of perceived high-risk pre-test probability for SMVP [21].

Recent studies have emphasized that strict blood pressure control during follow-up is a decisive modifiable factor influencing recurrence and outcome, reinforcing the need for structured post-discharge programs where imaging and prevention strategies are coordinated [8]. Formal consensus was modest and scenario-dependent, most notably in patients with deep ICH and SMVP = no. This reflects variation in pre-test thresholds for macrovascular disease, MRI/DSA access, and philosophy regarding the residual risk of a missed lesion versus the procedural burden of conventional invasive angiography. Modern diagnostic cerebral angiography conducted by neurointerventionalists carries a low, but non-zero, risk of neurologic complication (≈ 0.3% permanent deficit in contemporary series) [22].

Although CAA did not meet the threshold to enter our consensus tree as criterion, this omission may have influenced the apparent preference for MRI/MRA in older patients with lobar ICH. In everyday practice, older age and first manifestation of a lobar hemorrhage commonly lead to a CAA-oriented diagnostic work-up, and MRI is pivotal to identify hemorrhagic and white-matter markers incorporated in the Boston criteria v2.0 (e.g., superficial siderosis, multispot white-matter hyperintensity pattern, severe centrum semiovale perivascular spaces) [9, 23]. Consequently, some of the MRI/MRA utilization captured in our analysis likely reflects an implicit assessment for CAA that was not explicitly modelled within the consensus tree; we cannot quantify the extent of this effect from the available data. Recent evidence in lobar ICH survivors also suggests that CT-based markers may help rule-in probable CAA, but their absence is less useful to rule it out, supporting an ongoing role for MRI with MRA when CAA is suspected [24]. From our clinical point of view, future guidelines regarding the management and imaging follow-up of lobar ICH should include the suspicion of CAA in MRI as an important factor in guiding therapy and follow-up.

Our study has some limitations. Firstly, the number of participating centers, while pan-European and tertiary, was modest. Secondly, exclusion of certain criteria such as CAA and “clinical causes” improved comparability but may under-represent important patient subgroups. Thirdly, the survey question targeted non-surgically managed ICH, whose follow-up strategies would differ from patients requiring surgical or neurocritical care interventions. Finally, our analysis is based on expert-reported decision algorithms rather than patient-level data linked to clinical outcomes, which cannot determine whether the different proposed follow-up pathways translate into a significant improvement in long-term prognosis for patients.

## Conclusion

Across European academic centers, post-discharge imaging after non-surgically managed spontaneous ICH was variable, but coalesces around MRI/MRA at 6–12 weeks, with a combination of MRI/MRA with either CTA or DSA more often reported for cases with a credible macrovascular suspicion. Our findings describe current practice patterns, rather than clear clinical recommendations. Nonetheless, these could contribute towards standardization of follow-up management of patients with an acute ICH with clearly defined timelines and imaging procedures.

## Electronic Supplementary Material

Below is the link to the electronic supplementary material.

## Data Availability

Data generated or analyzed during this study are not publicly available in full, but are available from the corresponding author on reasonable request.
